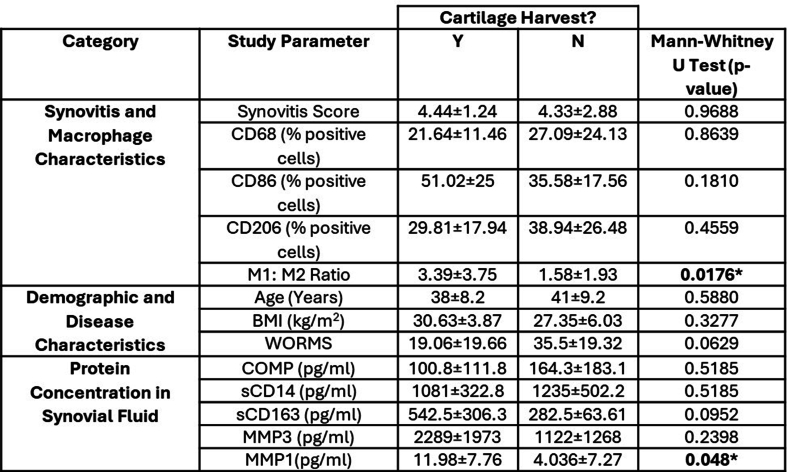# Increased synovitis and pro-inflammatory macrophage abundance are observed in the synovia of patients at risk of developing post-traumatic OA compared to those with established OA

**DOI:** 10.1016/j.ocarto.2025.100643

**Published:** 2025-07-18

**Authors:** Timothy Hopkins, John Garcia, Charlotte H. Hulme, Bernhard Tins, Jade Perry, Paul Jermin, Pete Gallacher, Andrew Barnett, Sally Roberts, Karina T. Wright

**Affiliations:** a Centre for Predictive In Vitro Models, Queen Mary University of London, London, UK; b Centre for Bioengineering, School of Engineering and Materials Science, Queen Mary University of London, London, UK; c Centre for Regenerative Medicine, School of Pharmacy and Bioengineering, Keele University, Keele, Staffordshire, UK; d Robert Jones and Agnes Hunt Orthopaedic Hospital, Shropshire, UK; e University College London, Department of Haematology, Cancer Institute, London, UK

**Keywords:** Synovium, Synovitis, Macrophages, Osteoarthritis, Patient stratification

## Abstract

**Objective:**

Inflammation of the synovium (synovitis) is implicated in the onset, progression and clinical manifestation of osteoarthritis (OA), although its prevalence at different stages of the disease has yet to be definitively established. Synovial macrophages play a central role in synovitis and can demonstrate pro- and anti-inflammatory phenotypes. The pervasiveness and variation in phenotypic identity of macrophages in early- and late-OA synovia is unclear. In the present study we investigated the frequency and severity of synovitis and assessed macrophage phenotypes in synovia from patients with high risk of developing PTOA (deemed early-OA) or late-OA.

**Design:**

Synovial samples were collected from patients undergoing cell therapy treatment for early-OA or arthroplasty for late-OA. Synovitis was assessed using a semi-quantitative, histological scoring system. Macrophage abundance and phenotypic characteristics were assessed by immunohistochemistry and image analysis. Study parameters were compared between the early- and late-OA groups and correlated with demographic and clinical information.

**Results:**

Synovitis was more prevalent and generally more severe in early-OA synovia compared to late-OA synovia (effect size; d ​= ​0.76). There were more macrophages overall (d ​= ​1.04), with more demonstrating markers characteristic of a pro-inflammatory (M1) phenotype (d ​= ​0.86), in the early-OA cohort. Synovitis severity was significantly correlated with the total number of macrophages (ρ ​= ​0.47), and with the presence of both M1 (ρ ​= ​0.65) and M2 (ρ ​= ​0.49) macrophage markers (M2 typically considered to indicate an anti-inflammatory or wound-healing phenotype).

**Conclusions:**

Our data suggest that synovial inflammation may play a greater role in the early stages of OA than in end-stage disease, and is at least partly mediated by synovial macrophages.

## 1 Introduction

Inflammation of the synovium (synovitis) is implicated in the onset and progression of osteoarthritis (OA) despite its traditional consideration as a non-inflammatory, degenerative disease [1,2]. The importance of synovitis in OA pathogenesis is supported by evidence from clinical studies that report associations between synovitis and joint space narrowing, cartilage loss and pain [1,3].

Synovitis is exemplified by cellular hyperplasia, tissue thickening and immune cell infiltration [1], accompanied by increased secretion of proinflammatory mediators, which attract circulating immune cells, and catabolic mediators, which degrade cartilage and remodel bone [4]. The products of cartilage degradation amplify synovial inflammation, creating a vicious cycle that, if left unchecked, leads to joint destruction [3]. The precise role played by synovial inflammation in contributing to the onset of OA, and in driving the radiographic and symptomatic progression of the disease is not fully characterised. This is hampered by considerable variation in the published literature in both patient cohort selection and in the method used to assess synovitis (e.g. histology [[5], [6], [7], [8]] or clinical imaging [9,10]), leading to reports of varying frequencies, even within similar cohorts. Further comparative research is required to fully characterise the pervasiveness of synovitis at different stages of disease progression. The first objective of the present study was to assess and compare the prevalence and severity of synovitis in two groups of patients at opposite ends of the knee OA spectrum: those at high risk, with cartilage defects (which if left untreated often progress to post-traumatic OA [11], thus representative of ‘early’-OA’ [12]), and the other with definite end-stage OA, ‘late-OA’, being treated by total knee replacement (TKR).

Another hallmark of synovitis is the accumulation of macrophages, driven by proliferation of the tissue-resident population and recruitment and differentiation of circulating monocytes [13,14]. In the inflamed joint, macrophages become activated and demonstrate divergent phenotypes [15]. These activations were traditionally described as polar, with those that are classically activated (M1), displaying a pro-inflammatory phenotype, and those that are alternatively activated (M2) displaying an anti-inflammatory, or reparative phenotype [15,16]. M1 polarised macrophages have been found to accumulate in OA synovium, and an M1-favoured imbalance of the ratio between M1:M2 macrophages, indicating a failure of the normal shift from inflammation to resolution, is reported to correlate with OA severity [17]. It is now accepted that macrophage phenotypes are far more heterogenous and less antithetical than previously thought, hence they can exist in a state of dynamic equilibrium with a broad spectrum of activation [18,19]. There has been little research investigating the phenotypical characteristics of synovial macrophages at different stages of OA progression. Thus, the second objective of this study was to assess synovial macrophages via immunohistochemistry in the two patient cohorts, to determine if there are key differences in the macrophage populations in synovia from patients with early-compared to late-stage OA.

## 2 Materials and methods

### 2.1 Patients and sample collection

A flowchart demonstrating the number of patients in each group, and subgroup, is shown in Fig. 1. Samples of infrapatellar fat pad (IFP)-associated synovia were collected during surgical intervention from a total of 30 patients in two cohorts. Due to the inherent differences in the surgical procedures by which the two cohorts were treated, the collection of synovial samples varied between cohorts. ‘Late-OA’ synovia were obtained by excision from waste IFP tissue collected from the knees of 15 patients undergoing TKR for the treatment of established or end-stage- OA. Additional synovial tissue samples were obtained by biopsy of IFP-associated synovia from 15 patients undergoing autologous cell implantation (ACI) for chondral or osteochondral defects of the knee. Without treatment, the patients in this cohort would be at risk of developing post-traumatic OA (PTOA), and are henceforth referred to as the ‘early-OA’ cohort. These patients were treated as part of an ongoing clinical trial (trial ID: ISRCTN98997175) that aimed to compare cell sources for autologous implantation [20]. All patients in the early-OA cohort underwent a two-stage surgical procedure. In the first stage, an arthroscopic surgery was performed, in which synovial fluid (SF) was collected, and the required cells were harvested: either chondrocytes from a knee hyaline cartilage biopsy and/or BMSCs from an iliac crest bone marrow biopsy, and culture-expanded (for further detail, see Richardson et al., 2017 ^20^). The cells were then implanted into the defect in the second stage, roughly 3–4 weeks later, at which time the IFP-associated synovial samples were collected by biopsy [20]. SF and blood plasma (by venepuncture) were also collected at this stage from the early-OA group for biomarker analysis, as previously described [21]. All patients provided informed consent, approved by National Research Ethics Committees 11/NW/0875 and 11/WM/0175.

**Fig. 1:**
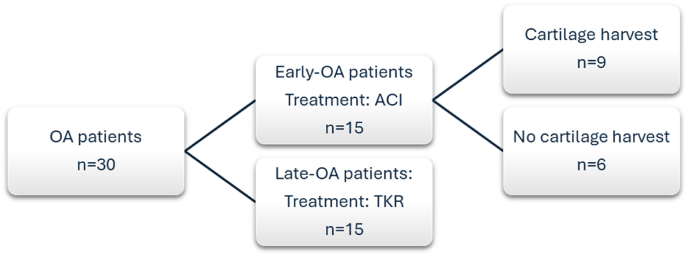
Study flowchart demonstrating group and subgroup sizes.

### 2.2 Assessment of disease severity by clinical imaging

The Kellgren-Lawrence (KL) score was used by an experienced radiologist to assess OA severity on pre-operative X-rays of late-OA patients [22]. The same radiologist used the Whole-Organ Magnetic Resonance Imaging Score (WORMS) to assess OA severity on pre-operative MRIs of early-OA patients [23].

### 2.3 Histological assessment of synovitis severity

Synovial tissue samples were processed according to established formalin-fixation and paraffin-embedding protocols, before sections were cut at a thickness of 5 ​μm and stained with haematoxylin and eosin (H&E) [24]. Synovitis was assessed using a validated categorical scoring system, with a total of 0 or 1 representing no synovitis, 2–4 low-grade synovitis and 5–9 high-grade synovitis [25].

### 2.4 Immunohistochemical assessment of macrophage characteristics

Proteins indicative of macrophage identity (CD68 – pan-macrophage marker [26]) and those associated with their functional phenotype (CD86 – indicating classical, M1 activation and CD206 – indicating alternative, M2 activation [27]), were assessed by immunohistochemical staining and subsequent semi-quantitative analyses. Immunohistochemistry was carried out according to protocols kindly supplied by Professor Gerjo van Osch (Rotterdam, Netherlands). A detailed protocol can be found in the supplementary materials and methods.

### 2.5 Image capture and analysis

Images were captured using a DS-FiL camera (Nikon Corporation, Tokyo, Japan) and processed using NIS-Elements BR software version 3.2 (Nikon Corporation) and FIJI-ImageJ (free software). The region of each H&E-stained synovial tissue section that corresponded with the highest (worst) synovitis score, was imaged for the proteins of interest and the number of positively stained cells (CD68^+^, CD86^+^ and CD206+) were counted and expressed as a percentage of the total nuclei counted on the corresponding image. A mean value for percentage positive cells was calculated for each protein of interest in each treatment group. A CD86+:CD206+ ratio, a proxy for the ratio between M1 and M2 polarised macrophages, was calculated for each sample.

### 2.6 Assessing the effect of cartilage harvest in the Early-OA group

In two of the three treatment arms of the clinical trial from which early-OA synovia samples were collected, the patients underwent a cartilage harvest. Study parameters were compared between early-OA patients who had, and had not, undergone cartilage harvest as part of their treatment. Additionally, several proteins of interest (cartilage oligomeric matrix protein (COMP), soluble CD14 (sCD14), sCD163, matrix metallopeptidase (MMP) −3 and −1) were quantified and compared in the SF collected from these patients by enzyme-linked immunosorbent assay (ELISA), as previously described [21]. Further details can be found in the supplementary materials and methods.

### 2.7 Statistical analyses

Statistical analyses were performed using GraphPad Prism (version 9.1.2; GraphPad Software, San Diego, USA). Normality was determined using the Shapiro-Wilk test. Mean synovitis, CD68^+^, CD86^+^, CD206+, WORMS, KL score, CD86+:CD206+ ratio and MMP1 concentration, were found to be non-normally distributed and therefore Mann-Whitney U tests were used to compare mean values between the early- and late-OA groups, and between the cartilage and non-cartilage-harvest groups of the early-OA cohort. Student's t-test for independent samples was used to compare demographic parameters between groups, which were found to be normally distributed. A p-value of ≤0.05 was considered statistically significant for these analyses. Correlation analyses (Spearman's rank correlation) were used to determine relationships between study parameters. A Bonferroni multiple testing correction (p<α/n-tests) was applied to the p-value of the correlation analyses to generate new statistical significance cut-offs, which are stated in individual results sections and figures. All data are presented as mean [95 ​% confidence intervals] unless otherwise stated. Effect sizes are presented as Cohen's d or Spearman's ρ, for difference and correlation analyses respectively.

## 3 Results

### 3.1 Patient demographics and disease severity

The mean age of the early-OA patients (39.2 [34.52, 43.88] years; range 21–51; Table 1) was significantly lower than that of the late-OA (67.3 [61.26, 73.27] years; range 46–86; p ​< ​0.001; d ​= ​3.33; Table 2). The early-OA group comprised 6 females and 9 males, and the late-OA group 8 females and 7 males. There was no significant difference in mean BMI (early-OA: 29.32 [26.58,32.05] kg/m^2^; Table 1; late-OA: 29.76 [28.05, 31.46] kg/m^2^; Table 2), which, for both groups, fell in the ‘overweight’ category according to NHS guidance. The mean WORMS for the early-OA cohort was 25.6 [14.24, 37.03]; range 4–67; Table 1). In the late-OA cohort, all patients had a KL score of 2 or greater (Table 2), which is the threshold considered to indicate radiographic OA (0 (0 ​%), 1 (0 ​%), 2 (20 ​%), 3 (33.3 ​%), 4 (46.7 ​%) [22].

**Table 1:** Demographic and disease severity information for early-OA cohort. Included are the significance values from statistical testing (Student's T or Mann-Whitney U) for differences in study parameters between patients that did, and did not, undergo a cartilage harvest as part of their treatment.

Group	Subgroup: Cartilage Harvest?	Age	BMI	Gender	Disease Severity (WORMS)
Early-OA	No	29	20.5	M	20
	No	34	23.239	M	30.5
	No	36	30.2	M	14
	No	46	34.181	M	67
	No	50	22.4	F	34
	No	51	33.58	M	47.5
	**Subgroup mean**	41	27.35	–	35.5
	**95 ​% CI**	[31.34, 50.66]	[21.02, 33.68]	–	[15.23, 55.77]
	Yes	21	29.98	F	65.5
	Yes	33	26.04	F	4
	Yes	35	36.26	F	9
	Yes	36	26.33	F	24
	Yes	37	28.473	M	21.5
	Yes	43	33.8	F	6
	Yes	43	27.55	M	7
	Yes	46	35.41	M	28.5
	Yes	48	31.79	M	6
	**Subgroup mean**	38.00	30.63	–	19.06
	**95 ​% CI**	[31.67, 44.33]	[27.65, 33.60]	–	[3.947, 34.16]
	**Test for differences (Student's T or Mann-Whitney U)**	0.588	0.3277	–	0.0629
Group mean		39.2	29.32	–	25.63
95 ​% CI		[34.52, 43.88]	[26.58, 32.05]	–	[14.24, 37.03]

**Table 2:** Demographic and disease severity information for late-OA cohort. Included are the significance values from statistical testing (Student's T) for differences in study parameters between the early- and late-OA cohorts.

Group	Age	BMI	Gender	Disease Severity (KL)
Late-OA	50	26.58	F	2
	46	29.76	F	3
	72	31.13	F	3
	68	29.74	F	3
	66	28.73	F	2
	60	29.24	F	4
	86	26.32	M	4
	76	36.9	F	4
	72	24.55	M	4
	68	27.99	M	2
	53	33	M	3
	71	33.26	M	4
	72	28.67	M	3
	78	31.06	F	4
	71	29.4	M	4
Group mean	67.27	29.76	–	3.27
95 ​% CI	[61.26, 73.27]	[26.58, 32.05]	–	[2.82, 3.71]
Student's T-test	<0.001	0.7716	–	–

### 3.2 Synovitis severity

The mean total synovitis score was significantly higher in the early-OA (4.40 [3.32, 5.48]) than in the late-OA group (2.27 [0.71–3.84]; p ​= ​0.027; d ​= ​0.76; Fig. 2). The modal total synovitis score for the cell therapy group was 3, compared to 0 in the TKR group. 60 ​% (n ​= ​9) of late-OA patients included in the study demonstrated no synovitis, compared to only 6.67 ​% (n ​= ​1) of the early-OA patients. A complete breakdown of the individual components of the synovitis scoring is shown in the supplementary materials (Supp Table 1).

**Fig. 2:**
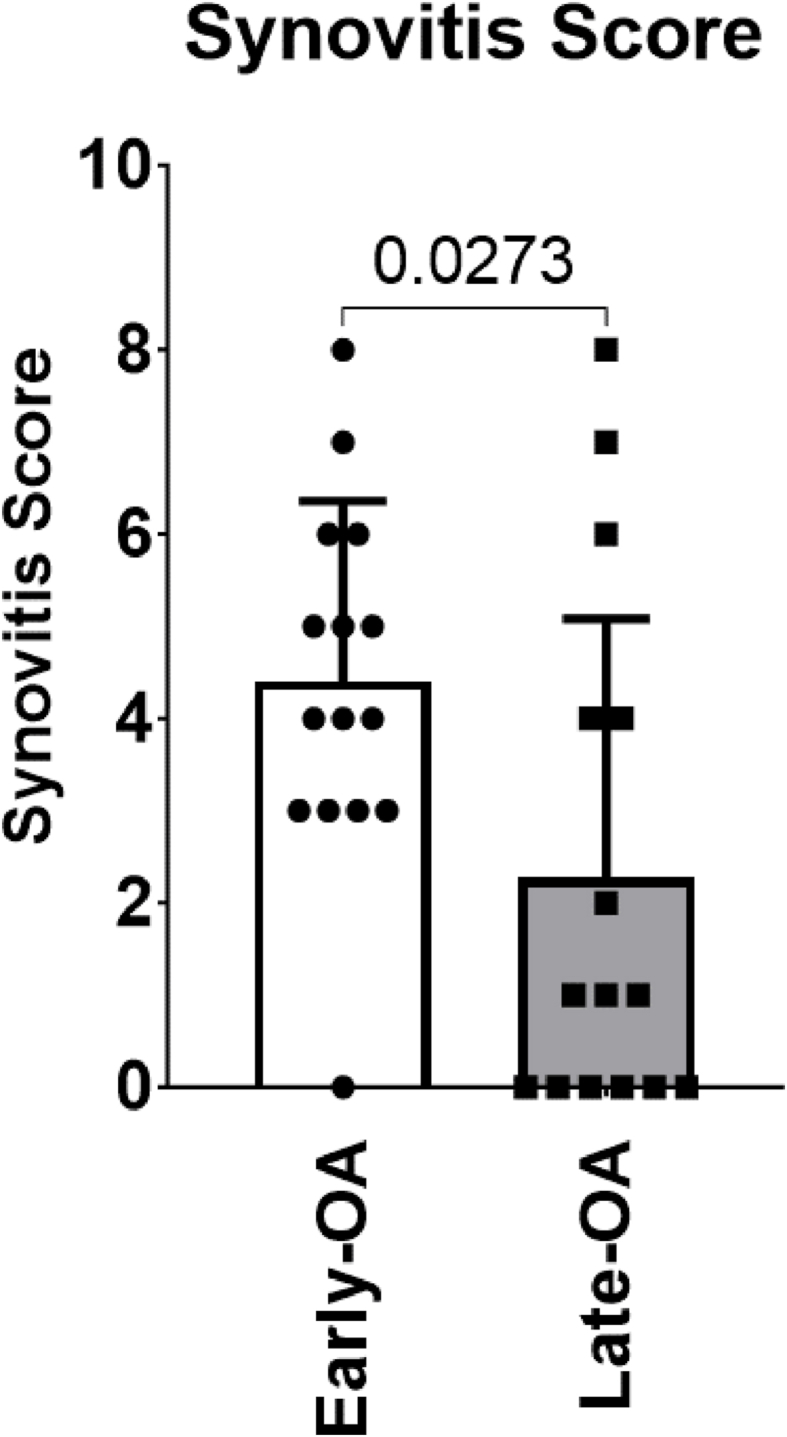
Mean total synovitis score (0–9) for the patients in the two treatment groups. Patients in the early-OA group had a significantly greater mean total synovitis score than did those in the late-OA group. Data is presented as mean ​± ​SD.

### 3.3 Immunohistochemical characteristics of synovial macrophages

Examples of the characteristic staining appearance of samples deemed to have high-grade and low-grade synovitis, based on the interpretation suggested by Krenn and colleagues [28], is shown for the early- and late OA groups (Fig. 3). The mean total number of cells was significantly higher in the early-OA synovia samples (561.5 [495.8, 627.1] cells) compared to the late-OA synovia samples (104.1 [59.54, 148.6] cells; p ​< ​0.0001; d ​= ​2.09; supplementary Figure 1). Mann-Whitney U-tests revealed that the mean percentage of CD68^+^ cells was significantly greater in the synovial tissue collected from early-OA patients (23.82 [14.38, 33.26] %) compared to that collected from the late-OA patients (6.1 [1.269, 10.95] %; p ​= ​0.0005; d ​= ​1.04; Fig. 4A). Similarly, a significantly higher number of cells were CD86^+^ in the early-OA synovial tissue (44.9 [32.11, 57.58] %) than in the late-OA synovial tissue (25.1 [7.305, 42.95] %; p ​= ​0.02; d ​= ​0.86; Fig. 4B). There was no significant difference in the mean percentage of CD206+ cells (Fig. 4C). The CD86+:CD206+ ratio was significantly greater in the synovial tissue collected from early-OA patients (2.7 [0.894, 4.435]) compared to that collected from late-OA patients (1.2 [0.054–2.354]; p ​= ​0.016; d ​= ​0.47; Fig. 4D).

**Fig. 3:**
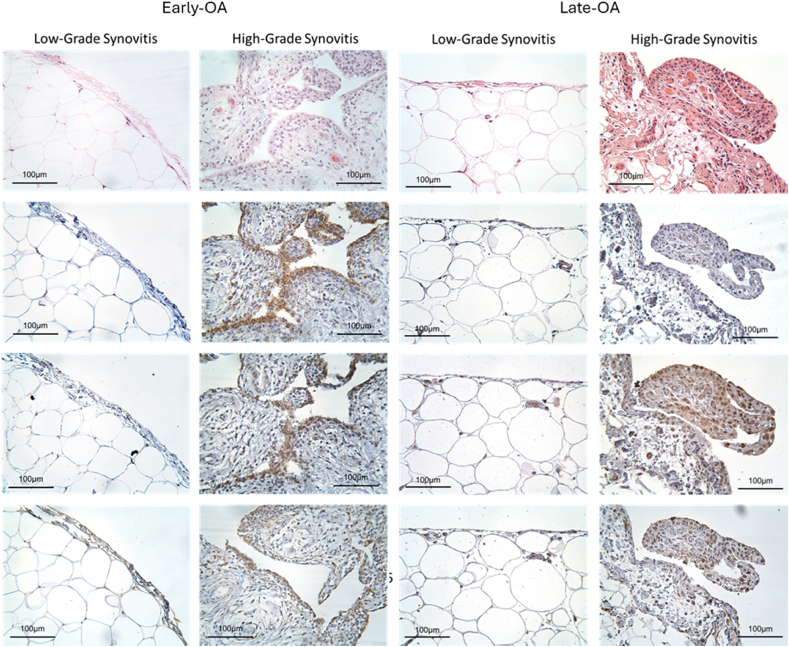
Characteristic staining profiles of synovia isolated from early-OA and late-OA patients. From top to bottom, images demonstrate histological staining patterns for H&E and immunohistological staining patterns for CD68, CD86 and CD206. The sample in the left-hand column for each cohort was determined to exhibit low-grade synovitis and that in the right-hand column high-grade. Scale bar ​= ​100 ​ ​μm.

**Fig. 4:**
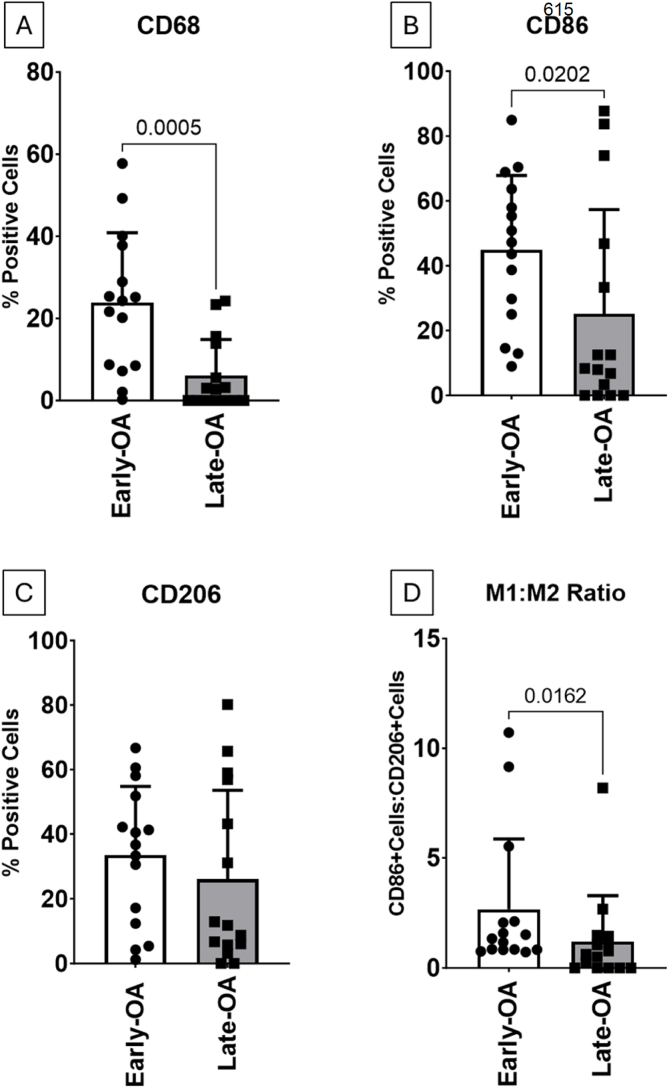
Comparative mean percentage of total cells that positively stained for (A) CD68, (B) CD86 and (C) CD206 in synovia from the early-OA and late-OA cohorts. Also shown in (D) is a comparative mean ratio of CD86 positive cells to CD206 positive cells representing a ratio of M1:M2 macrophages) between patients in the two cohorts. Data is presented as mean ​± ​SD and P values ​< ​0.05 are shown.

### 3.4 Identifying relationships between study parameters

Correlation analyses were initially carried out on the full dataset (n ​= ​30) to identify relationships between individual study parameters (Table 3). A Bonferroni correction for multiple comparisons, where p ​< ​alpha/n tests, was applied (in this case: p ​< ​0.05/21 ​= ​0.00238). Total synovitis score was positively correlated with the percentage of CD68^+^ (ρ ​= ​0.54, p ​= ​0.002), CD86^+^ (ρ ​= ​0.67, p ​< ​0.001) and CD206+ (ρ ​= ​0.56, p ​= ​0.001) cells. Subsequently, a multiple linear regression model was utilised to identify individual predictors of total synovitis score, with the additional parameters: affected knee (right or left), sex (male or female) and treatment group (ACI or TKR). There were no significant predictors of total synovitis score identified when assessing the full dataset.

**Table 3:**
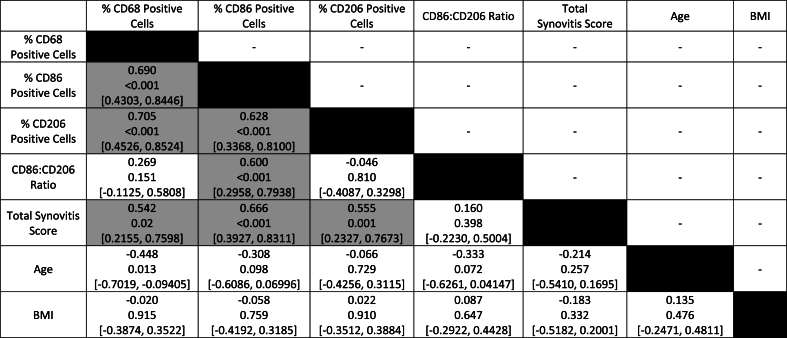
Correlation matrix of the full dataset (n ​= ​30) to highlight relationships between study parameters. Data is presented as Spearman's ρ and p-values in parentheses. Shaded boxes indicate statistically significant correlations.

Correlation and subsequent multiple linear regression analyses were then carried out on the early-OA (Supplementary Table 2) and late-OA (Supplementary Table 3) patient cohorts individually. A Bonferroni correction for multiple comparisons was applied (p ​< ​0.05/28 ​= ​0.00179). Disease severity scores (WORMS and KL) were included as additional parameters in these correlation analyses. In both individual groups, the percentage of CD68^+^ cells was significantly correlated with the percentage of CD206+ cells. In the late-OA group, total synovitis score was negatively correlated with BMI (ρ ​= ​−0.78, p ​= ​0.001). Additional multiple regression analyses were also carried out to identify predictors of radiographic disease severity for each cohort. There were no significant individual predictors of disease severity in either the early-OA or late-OA group.

### 3.5 Assessing the effect of cartilage harvest in the Early-OA group

**Fig. 5:**
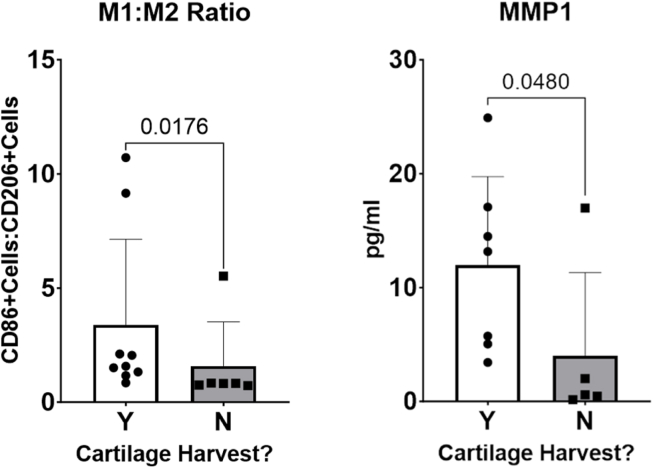
Comparative M1:M2 ratio, and MMP1 concentration in the synovial fluid of patients who did (Y) and did not (N) undergo a cartilage harvest as part of their treatment.

The study parameters were then compared between patients within the early-OA group who had (n ​= ​9) and had not (n ​= ​6) undergone a cartilage harvest as part of their treatment (supplementary Table 4), as this procedure has been shown to produce an inflammatory response in a subset of patients [[29], [30], [31]]. There was no difference in age, BMI or disease severity (WORMS) between groups, as expected due to the randomisation of patients to the treatment groups (supplementary Table 4). Neither was there a significant difference in mean synovitis score, or in the percentage CD68^+^, CD86^+^ or CD206+ cells in the synovia from the two groups. However, the CD86+:CD206+ ratio was found to be significantly greater in the cartilage harvest group (3.386 [0.50, 6.27]) than in the non-harvest group (1.582 [−0.4471, 3.611]; p ​= ​0.0176; d ​= ​0.48; Fig. 5). There was also a significant difference in MMP1 concentration measured in the synovial fluid of the patients who underwent a cartilage harvest (11.98 [4.812–19.16] pg/ml) compared to those who did not (4.036 [−4.994,13.07] pg/ml; p ​= ​0.048; d ​= ​2.71; Fig. 5). There was no significant difference in the concentration of any of the other proteins quantified in the synovial fluid.

## 4 Discussion

Despite the reported prevalence of synovial inflammation in OA, it remains to be determined whether its presence represents a subgroup of the disease or a ubiquitous feature of disease progression. This study highlights considerable heterogeneity in the presence and severity of synovitis in early- and late-OA, with synovial samples in both groups demonstrating no, low- and high-grade synovitis. However, a greater proportion of the late-OA synovia exhibited no synovitis, and that synovitis was more prevalent, and on average, more severe in synovia from the early-OA cohort. The majority of earlier studies report that synovitis is more prevalent in later-OA than at earlier stages [7,[32], [33], [34]]. However, one study corroborates our results, showing increased markers of synovial inflammation (mononuclear cell infiltration, expression of inflammatory mediators, blood vessel infiltration) in early-OA synovia compared to late-OA synovia [35]. Interestingly, interrogation of the individual components of the synovitis scoring system revealed that the difference in synovitis between the two OA groups was largely driven by the difference in the ‘enlargement of the synovial lining layer’ component. The mean score for this component was significantly greater in the early-OA cohort than in the late-OA cohort (2.07 [1.62, 2.51] vs 0.67 [0.17, 1.16]; p ​= ​0.001) (Supplementary Table 1). There was no significant difference in either of the other two parameters that comprise the score. While of interest, the separation of scoring system components is not recommended by its authors and should be interpreted cautiously.

Further contention is evident in the reported prevalence of synovitis between groups of patients at seemingly the same stage of OA progression. For late-OA groups, in which synovial samples were collected during arthroplasty, the reported prevalence of synovitis includes 100 ​% [36], 93.3 ​% [37], 57 ​% [5] and 28 ​% [6]. In comparison, we found that synovitis was present in 40 ​% of late-OA synovia. Similarly, studies of synovitis in early-OA cohorts, with samples collected by arthroscopy or during meniscectomy, reported a synovitis prevalence of 55 ​% [7], 57 ​% [38] and 43 ​% [8], whereas we identified synovitis in 93.3 ​% of synovia collected from our early-OA cohort. This disparity between our findings and those of previous works could be due to the surgical procedure which the patients in the early-OA cohort of the present study underwent. The synovial samples in this cohort were collected during the second stage of the two-stage procedure, which routinely takes place around 3 weeks after the first stage. Thus, the increased presence of synovial inflammation in this cohort could be an acute response to the first stage surgery (an arthroscopic viewing of the joint and in some cases a cartilage harvest). However, there was no difference in synovitis prevalence and severity observed between those that underwent a cartilage harvest, and those that didn't (the most invasive part of this procedure), therefore it could be asserted that the response to only an arthroscopic viewing of the joint is unlikely to have resulted in an acute synovial response in almost all early-OA patients and the observed synovitis therefore is more likely reflective of their baseline OA phenotype.

A statistically significant correlation (ρ ​= ​0.542, p ​= ​0.002) between total synovitis score and the mean percentage of CD68^+^ cells was observed across all patients (n ​= ​30) in this study. This finding was unsurprising as synovial macrophages, indicated by CD68 positivity, are known to be largely responsible for driving synovial inflammation [14]. Increased synovial inflammation, total macrophage number and macrophages with markers indicative of M1 activation (CD86^+^), were identified in the early-OA, compared to the late-OA cohort. There is some debate regarding the polarisation status of activated macrophages in late-OA tissue. While Griffin and colleagues reported that both M1 and M2 macrophages are present in end-stage OA synovia [39], others have reported the accumulation of M1, but not M2, macrophages [40]. The variability in the literature reflects the complexity of macrophage activation, which is accepted to be far more spectral than the M1/M2 terminology and earlier concepts suggest.

In a study by Wu and colleagues, in which macrophages were selectively depleted in a murine OA model (destabilisation of the medial meniscus), resulting in a reduction of both M1 and M2 macrophages, but greater synovitis and systemic inflammation [41]. This finding suggests that macrophages may not be the sole mediator of synovial inflammation. FLS, the other major resident cell type in the synovium, are also known to play a critical role in moderating synovial inflammation [42]. In the inflamed synovium, FLS rapidly proliferate, leading to considerable tissue hyperplasia [1], which was clearly evident in synovial sections studied in the present study and reflected in the significantly higher total cell number in the early-OA synovia. Previously published work has highlighted the important role of FLS in the onset and symptomatic presentation of OA [43,44]. Further assessment of FLS characteristics and contribution to synovitis is required, but was beyond the scope of the present study. The authors are, however, currently working on the development of organ-on-a-chip models of the human synovium, incorporating multiple cell types for improved disease modelling and therapeutic screening [45].

As part of their cell therapy treatment, some of the early-OA patients underwent a cartilage biopsy. It has been previously reported that a proportion of patients who undergo a cartilage harvest as part of their treatment have a marked proinflammatory and catabolic response to the procedure, detectable in their SF [[29], [30], [31]]. However, there was no difference in synovitis prevalence or severity in the cartilage harvest subgroup of early-OA patients, and therefore the procedure had no impact on synovitis. There was a slight increase in the percentage of CD86^+^ cells and a slight decrease in the percentage of CD206+ cells in the cartilage harvest group, that, while not statistically significant, did result in a statistically significant difference in CD86+:CD206+ ratio. Additionally, the concentration of MMP1 measured in the SF, was significantly higher in the cartilage harvest group, suggesting an inflammatory/catabolic response to the biopsy, which corroborates previous findings in some patients and may aid in patient stratification [31]. While of interest, the small sample size in the present study represents a limitation of the present work and these investigations would benefit from larger patient cohorts.

While the method of sampling varied between the early- and late-OA cohorts due to inherent differences in the surgical procedures, consistency of sampling location was ensured in both cases by collecting IFP-associated synovium. However, it is possible that, regardless of the method employed, the discrete nature of methods for measuring synovitis at single timepoints is inappropriate for the assessment of a highly dynamic process such as synovial inflammation. Inflammation is a critical mechanism of innate immunity, representing one of the first steps in a healing process in response to tissue injury and infection. It may be the case that in some patients, synovial inflammation will simply resolve, and it is not the synovial inflammation itself that is the issue, but the lack of its resolution that it then becomes chronic in nature. Therefore, our study, and those that came before it, may be detecting synovitis that might otherwise have resolved, making it even harder to determine its role in the disease process. In support of the hypothesis that properly regulated synovial inflammation is part of the normal immune response, a previous post-mortem study reported a prevalence of synovitis of 11 ​% in patients with no OA history or pain [3]. New, non-invasive methods to quantify synovitis *in vivo*, such as dynamic contrast-enhanced magnetic resonance imaging [46] may provide the means to assess synovitis over the course of OA disease progression and provide further clarity.

In this exploratory study, the authors used markers indicative of the dichotomous, M1/M2 paradigm of macrophage polarisation. The authors appreciate that synovial macrophage identity is far more complex and less antithetical than this classification implies. Previously published work has highlighted significant complexity in synovial macrophage phenotypes, using more sophisticated methods. In separate studies, Chou and colleagues (2020) and Huang and colleagues (2021) used single-cell RNA sequencing to characterise cellular subsets in OA synovium, identifying discrete subgroups [42,47], as well as a transitional subgroup [42]. Several studies have also shown that there is significant overlap in M1/M2 activation, which represent the polar extremes of a spectrum of macrophage activation, and that OA synovial tissues express a mixed pattern of both markers [[48], [49], [50]]. Mimpen and colleagues also highlighted that macrophage phenotypes vary by location within the synovium [49]. While the value of the present study lies in the comparison of early- and late-OA synovia, further characterisation of the macrophage population would add value to future studies.

To conclude, an improved understanding of the role of macrophages and how their function is altered could further our understanding of the aetiopathogenesis throughout the osteoarthritic pathway in both post-traumatic and idiopathic groups. By doing so, we can perhaps decide more accurately which patients are at higher risk of rapid disease progression, as well as indicating potential therapies, for example via encouraging MSC modification of macrophage behaviour. We are currently developing organ-on-a-chip based models of the human synovium and cartilage, using primary cells, to further investigate the role played by synovial inflammation in the onset and progression of osteoarthritis, and to seek to stratify patients based on this interaction.

## Author contributions

Conception and design: TH, KTW, JG, CH, SR. Analysis and interpretation of the data: TH, KTW, JG, CH, SR, BT, JP. Drafting of the article: TH, KTW, CH, SR. Critical revision of the article for important intellectual content: TH, KTW, CH, SR. Final approval of the article: TH, KTW, JG, CH, BT, JP, PJ, PG, AB, SR. Provision of study materials or patients: TH, JG, CH, PJ, PG, AB. Obtaining of funding: KW, SR, JG. Collection and assembly of data: TH, JG, JP, BT, CH.

## Role of the funding source

The funder (Orthopaedic Institute Ltd) had no involvement in the study design, data collection, analysis and interpretation of data; in the writing of the manuscript; and in the decision to submit the manuscript for publication.

## Declaration of competing interest

The authors declare no conflict of interest.

## Figures and Tables

**figs1:**
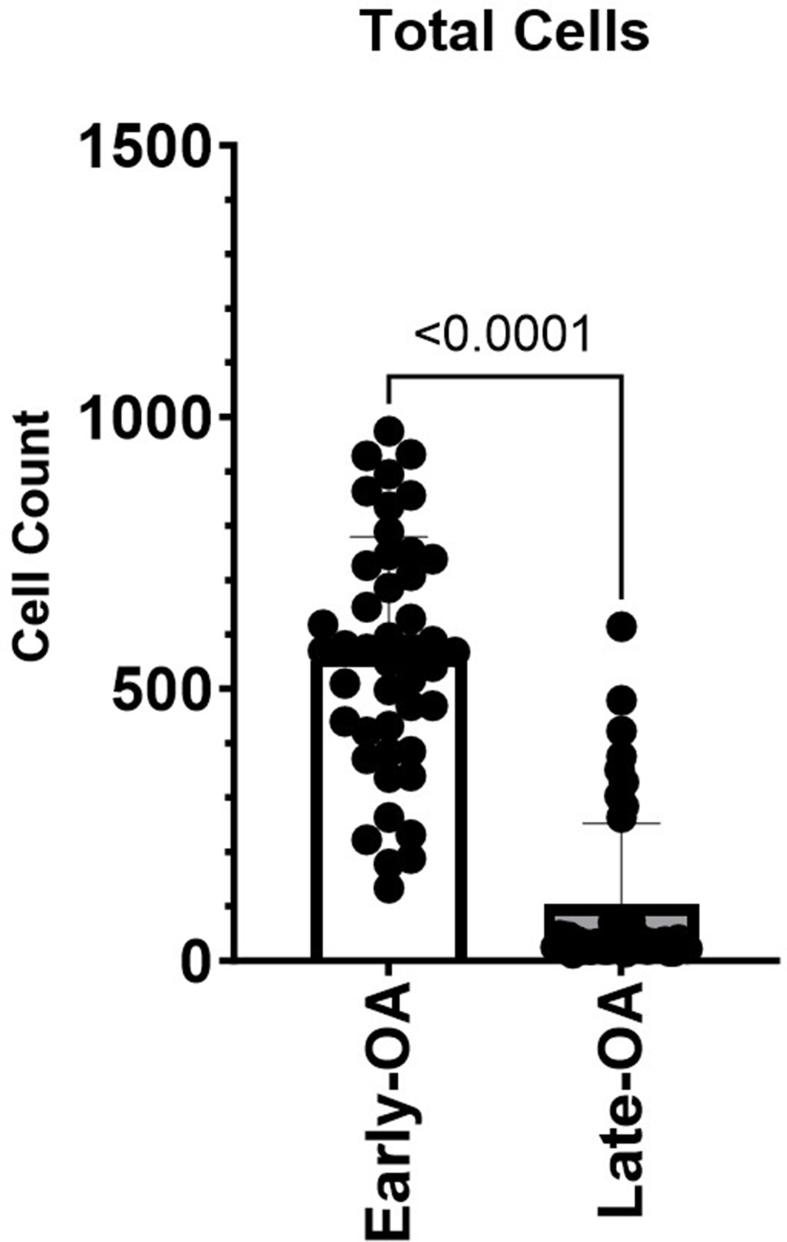


**figs2:**
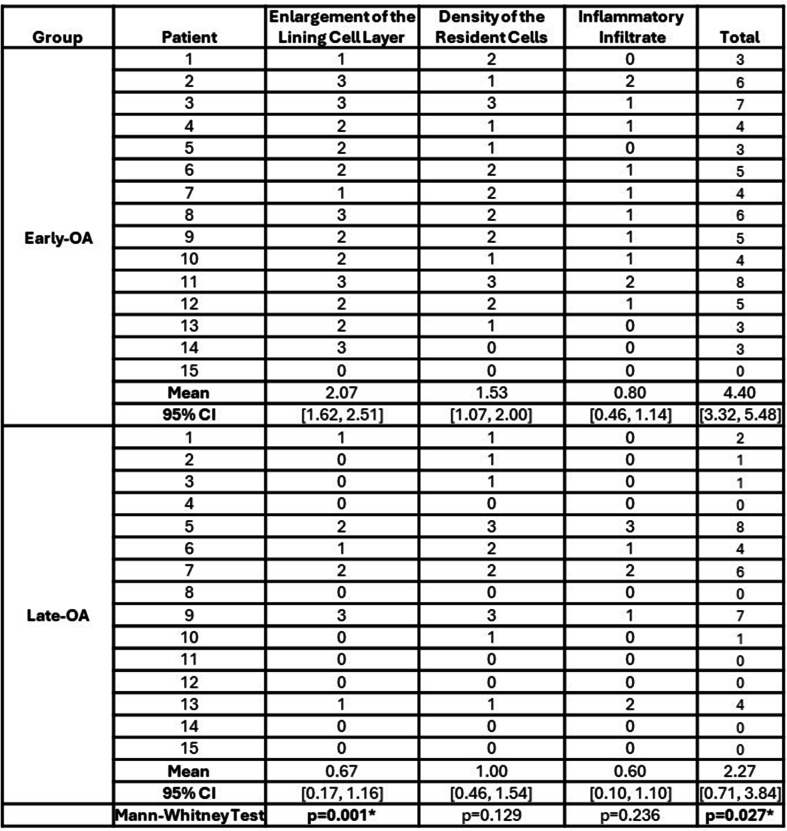


**figs3:**
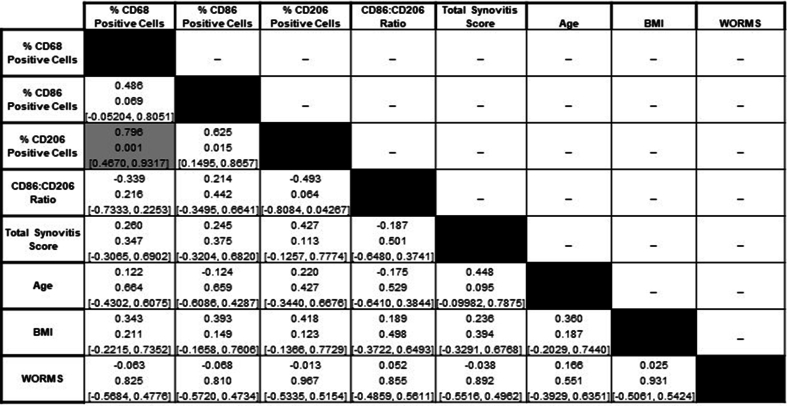


**figs4:**
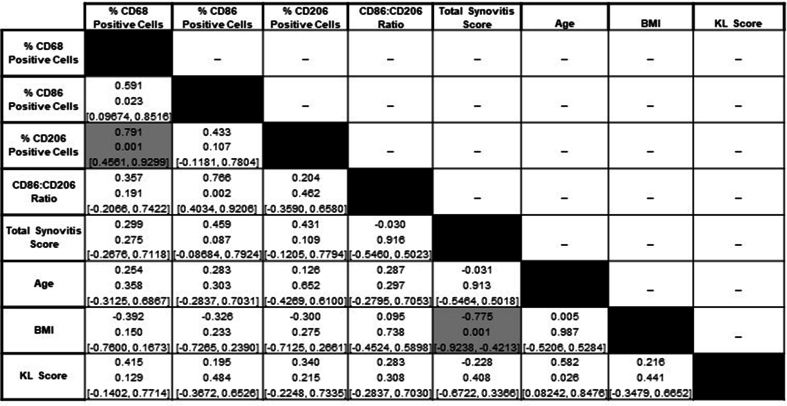


**figs5:**