# Supplementary material for: Increased synovitis and pro-inflammatory macrophage abundance are observed in the synovia of patients at risk of developing post-traumatic OA compared to those with established OA
Source: Osteoarthr Cartil Open. 2025 Jul 18;7(3):100643. doi: 10.1016/j.ocarto.2025.100643 (PMC12309280; doi:10.1016/j.ocarto.2025.100643)
Supplement: Multimedia component 1 [file mmc1.docx]

## Supplementary Information

1. Supplementary Materials and Methods

Immunohistochemical Assessment of Macrophage Characteristics: Heat-induced epitope retrieval was performed using either sodium citrate buffer (CD68 and CD206; pH6.0, 10mM sodium citrate, 0.05% Tween 20 (both Sigma-Aldrich)) or Tris-EDTA buffer (CD86; pH9.0, 10mM Tris base, 1mM ethylenediaminetetraacetic acid (EDTA), 0.05% Tween 20 (all Sigma-Aldrich)). Synovial sections were immersed in the indicated buffer, heated to 95°C in a water bath for 20 minutes and then incubated at room temperature for 15 minutes. Slides were washed three times with 0.1% Tween-20 in Dulbecco’s phosphate buffered saline (PBS, Gibco, Loughborough, UK). The wash step was repeated between all subsequent steps, which were carried out at room temperature. Non-specific binding of the primary antibodies was blocked by incubation with normal goat serum (Vector Laboratories, Upper Heyford, UK) for 30 minutes. Slides were then incubated with rabbit monoclonal antibodies against CD68 (clone EPR20545; Abcam, Cambridge, UK), CD86 (clone EP1158Y; Abcam) or a rabbit polyclonal antibody against CD206 (Abcam) for 60 minutes, then incubated with a biotinylated goat anti-rabbit secondary antibody (Vectastain® Elite ABC kit, Vector Laboratories) for 30 minutes. Negative controls were established using an isotype-matched IgG (cat. No: I-100-5; Vector Laboratories) in place of the primary monoclonal antibodies, and normal rabbit serum (cat. No.: ab7487; Abcam) in place of the primary polyclonal antibody. Endogenous peroxidase activity was blocked using 0.3% hydrogen peroxide in methanol, for 30 minutes. Tissue sections were subsequently incubated for 30 minutes with the avidin-biotin complex (ABC) reagent (Vectastain® Elite ABC kit) to enhance labelling. ImmPACT® DAB (diaminobenzidine) peroxidase substrate (Vector Laboratories) was applied for 6 minutes to reveal antibody staining. Sections were counterstained with haematoxylin and dehydrated through a series of increasing concentrations of IPA before being cleared in xylene and mounted in Pertex (CellPath, Newtown, UK).

Assessing the Effect of Cartilage Harvest in the Early-OA Group: These proteins (and assay kits) were Cartilage Oligomeric Protein (COMP (BioVendor Laboratory Medicine), soluble Cluster of Differentiation 14 (sCD14), sCD163, Matrix Metalloproteinase 3 (MMP3) (all Biotechne Quantikine ELISAs) and MMP1 (Biotechne Duo-Set). Samples were diluted (over and above dilution for lavage) at 1:3000 (COMP); 1:12 (sCD163); 1:200 (sCD14); 1:3 (MMP1); 1:100 (MMP3) and analyses performed according to manufacturers’ instructions. The concentration of each protein was normalised to the dilution factor of the SF (due to lavage), which was determined by measuring urea concentration in the matched plasma and SF samples (QuantiChrom Urea Assay Kit, Universal Biologicals).
