# Supplementary material for: Increased synovitis and pro-inflammatory macrophage abundance are observed in the synovia of patients at risk of developing post-traumatic OA compared to those with established OA
Source: Osteoarthr Cartil Open. 2025 Jul 18;7(3):100643. doi: 10.1016/j.ocarto.2025.100643 (PMC12309280; doi:10.1016/j.ocarto.2025.100643)
Supplement: Multimedia component 2 [file mmc2.docx]

## Supplementary Figure Legends

1. Supplementary Table 1: Individual components of synovitis scoring system in the early- and late-OA cohorts.
2. Supplementary Figure 2: Mean total cell number in the Early- and Late-OA cohorts.
3. Supplementary Table 2: Correlation matrix of the cell therapy-treated cohort (n=15) to highlight relationships between study parameters. Data is presented as Spearman’s ρ and p-value (in parentheses). Shaded boxes indicate statistically significant correlations.
4. Supplementary Table 3: Correlation matrix of the TKR-treated cohort (n=15) to highlight relationships between study parameters. Data is presented as Spearman’s ρ and p-value (in parentheses). Shaded boxes indicate statistically significant correlations.
5. Supplementary Table 3: Comparison of study parameters between the cartilage harvest and non-cartilage harvest groups of the early-OA cohort.
